# Double Tubal Pregnancy—Both Tubes Ruptured

**Published:** 1916-02

**Authors:** 


**Double Tubal Pregnancy—Both Tubes Ruptured.** D. H. Galloway, Boswell, N. W., reports such a case in Am. Med., Dec. Recovery, after operation.

